# Synergistic activity of fosfomycin and flucloxacillin against methicillin-susceptible and methicillin-resistant* Staphylococcus aureus:* in vitro and in vivo assessment

**DOI:** 10.1007/s00430-025-00841-3

**Published:** 2025-06-21

**Authors:** Alina Nussbaumer-Pröll, Markus Obermüller, Matthias Weiss-Tessbach, Sabine Eberl, Markus Zeitlinger, Bernd Matiba, Christian Mayer, Manuel Kussmann

**Affiliations:** 1 Department of Clinical Pharmacology, Medical University of Vienna, Vienna, Austria; 2 Department of Medicine I, Division of Infectious Diseases and Tropical Medicine, Medical University of Vienna, Währinger Gürtel 18-20, 1090 Vienna, Austria; 3 InfectoPharm Arzneimittel und Consilium GmbH, Von-Humboldt-Strasse 1, 64646 Heppenheim, Germany

**Keywords:** Combination therapy, MRSA, Antimicrobial resistance, Experimental model, In vivo synergy

## Abstract

Fosfomycin (FOF) exhibits broad-spectrum antimicrobial activity, and is mainly used in combination therapy. Previous in vitro studies have shown synergistic effects of FOF in combination with flucloxacillin (FLX) against *Staphylococcus aureus* isolates. This study aims to validate these findings in vitro and investigate the synergistic effect in an in vivo* Galleria mellonella* model. Five methicillin- and FOF-susceptible isolates (ATCC-29213 & 4 clinical isolates); one methicillin- and FOF-resistant strain (DSMZ-23622) and four methicillin-resistant and FOF-susceptible strains (ATCC-33592 & 3 clinical isolates) were tested with checkerboard assays to assess synergism. Time-kill curves were generated for two MSSA (ATCC 29213 and 231/20) and two MRSA strains (ATCC 33592 and DSMZ 23622). The in vivo efficacy of FOF and/or FLX was evaluated by a *G. mellonella* survival assay and by determining the total bacterial count (TBC) in hemolymph. Checkerboard assays revealed additive or indifferent effects, with some indicating synergism. Time-kill curves demonstrated higher reduction in TBC with combination therapy compared to monotherapy. In vivo, the combination therapy showed the greatest reduction of TBC in larval haemolymph compared to monotherapy, and the survival assay showed highly synergistic activity of FLX plus FOF against MRSA (ATCC-33592) and MSSA (ATCC 6538), resulting in an average reduction in mortality of 48 and 40%, respectively, compared to monotherapies. Therefore, FOF plus FLX could be an alternative for the calculated or definitive treatment of *S. aureus* infections without antimicrobial susceptibility results or even for salvage therapy of MRSA infections after treatment failure or necessary discontinuation of classical MRSA drugs.

**Graphical abstract:**

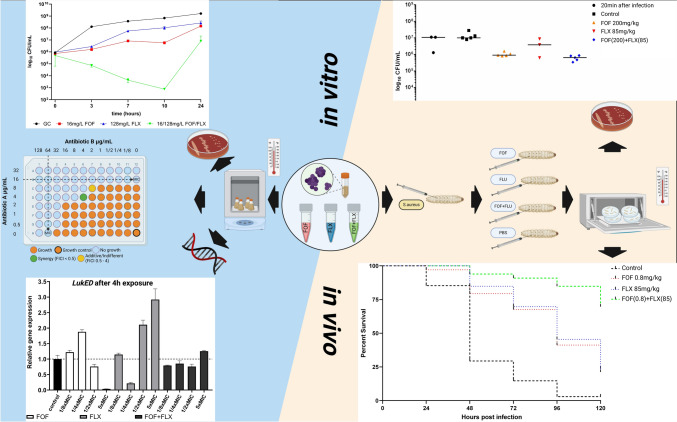

**Supplementary Information:**

The online version contains supplementary material available at 10.1007/s00430-025-00841-3.

## Introduction

Antibiotic misuse has led to bacterial multi-drug resistance, a significant health challenge in the twenty-first century [1]. Infections with multi-drug resistant (MDR) bacteria cause approximately 25,000 deaths annually in the European Union, with methicillin-resistant *Staphylococci* posing particular treatment challenges [2, 3]. Fosfomycin (FOF), effective against a wide range of bacteria, is recommended for combination therapy to prevent rapid selection of resistant mutants [4, 5]. Numerous studies have shown synergistic activity between FOF and various beta-lactam and non-beta lactam antimicrobials against methicillin-susceptible and methicillin-resistant *Staphylococcus aureus* (MRSA) strains, including combinations with cefpirome, cefazolin, cefotaxime, linezolid, daptomycin and oxacillin [3, 4, 6–8]. Furthermore, the combination of FOF with various antibiotics in vitro and in vivo has been thoroughly discussed in the systematic review by Antonello et al., describing the potential of combination therapy against *Pseudomonas aeruginosa*, *Enterococcus spp*., and *Acinetobacter spp*. [9].

In line with this, clinical trials investigating FOF plus imipenem as therapy for complicated bacteraemia and infective endocarditis caused by MRSA also provided at least a proof of concept warranting further research in this specific area. [10, 11]. Another promising combination with FOF might be flucloxacillin (FLX), as Landersdorfer et al*.* demonstrated that FLX achieves a high probability of target attainments against *S. aureus*, specifically methicillin-sensitive strains (MSSA), at standard doses [12]. Furthermore, considering the low concentrations required to achieve the MIC-50% (0.2 µg/mL) and the MIC-90% (0.39 mg/L) values for MSSA, the combination of FLX with FOF might resemble a promising treatment option [13].

Thus, we set out to assess the synergistic effects of FLX and FOF against various *S. aureus* strains, including methicillin-resistant isolates.

## Materials and methods

### Bacterial strains

In this study, eleven *S. aureus* isolates including six methicillin- and FOF-susceptible (ATCC-29213, ATCC 6538 and four clinical isolates), one methicillin- and FOF-resistant (DSMZ-23622), and four methicillin-resistant and FOF-susceptible (ATCC-33592 and three clinical isolates) were used. All clinical isolates were routinely obtained and identified from positive blood cultures and provided by the Department of Clinical Microbiology of the General Hospital in Vienna.

### Antimicrobial susceptibility and synergy testing

The minimum inhibitory concentrations (MICs) of FLX and FOF were determined by broth microdilution method using cation-adjusted Mueller–Hinton broth (CA-MHB) as recommended by the European Committee on Antibiotic Susceptibility testing (EUCAST) [14]. In antibiotic susceptibility testing with FOF, the presence of glucose-6-phosphate (G6P) in MHB is mandatory as it mimics physiological conditions and ensures that the activity of FOF is accurately assessed in vitro. Without G6P, the bacteria may not take up sufficient amounts of FOF, leading to artificially high MICs and potentially misleading results [15]. Thus, as in FOF testing G6P addition to MHB is mandatory and FLX is not routinely tested in MHB with G6P, additional MICs were conducted with and without G6P to evaluate any impact that it might have on FLX activity.

Synergy testing was performed using a slightly modified checkerboard assay. For this purpose, 96-well U-bottom microtiter plates with a final inoculum of approximately 5 × 10^5 CFU/mL and a final volume of 200 µL per well were used. Assessment was performed after an incubation period of 18–24 h at 36 °C (± 1 °C). Plates were assessed after an incubation period of 18–24 h at 36 °C (± 1 °C), and the results obtained were used to calculate fractional inhibitory concentration indices (FICI).

The calculation of the FICI was done by the formula (MIC of A in combination with B / MIC of A) divided by (MIC of B in combination with A / MIC of B), resembling A as FOF and B as FLX. FICI results were interpreted as synergism ≤ 0.5, > 0.5–4 = no interaction, and > 4 antagonism.

The susceptible breakpoint index (SBPI) evaluates the MICs of agents when tested in combination relative to their individual susceptibility breakpoints, and may better reflect clinical applicability than the traditional FICI. [16]. For calculation of the SBPI the clinical breakpoint of 32 mg/L for intravenous FOF and the PK/PD breakpoint (fT > MIC ≥ 50% with 2 g q4h) of 1 mg/L for FLX were used, both obtained from the EUCAST. Calculation of SBPI = (susceptible breakpoint A/combination MIC A) + (susceptible breakpoint B/combination MIC B). An SBPI ≥ 2 indicates that the combined MICs of the tested antimicrobials are equally or lower than their respective breakpoints. Thus, the greater the SBPI value, the more effective the antimicrobial combination. All experiments were performed up to 10 times and at least 5 times.

### Time kill curves (TKC)

Time Kill Curves were conducted with two MSSA (ATCC-29213 and MSSA 231/20) and two MRSA (23,622 DSMZ (FOF R) and ATCC 33592 (FOF S)) strains with concentrations evaluated by the checkerboard assays showing synergy or additive behavior. Antibiotics were tested alone and in combination. TKC were performed in triplicates over 24 h in a water bath at 37 °C under aerobic conditions with MHB supplemented with 25 mg/L G6P in 14 mL falcon tubes. The bacterial suspension was adjusted to 1.5 × 10^8 cells/mL in NaCl, corresponding to a McFarland standard of 0.5, and was added to the test tubes at a final concentration of 1.5 × 10^6 colony forming units (CFU)/mL. Samples were drawn at time point 0 (before the addition of antibiotics) and then at 4, 7, 10 and 24 h. Subsequently, seven serial dilution steps were carried out in 96-well microtiter plates filled with 0.9% NaCl. Twenty µL were then dropped onto Columbia blood agar plates and incubated at 37 °C under aerobic conditions for 24 h. After incubation the CFU were counted, and the CFU/mL was calculated taking the dilution steps into account.

### In vivo* galleria mellonella survival assay*

Three *S. aureus* isolates, two methicillin-sensitive (ATCC 29213, ATCC 6538) and one methicillin-resistant (ATCC 33592), were used in the *G. mellonella* survival assay. Bacterial inocula were prepared by diluting 24 h liquid cultures with fresh tryptic soy broth (TSB) followed by incubation on an orbital shaker at 36–37 °C to obtain bacteria in the exponential growth phase with a bacterial load causing the targeted mortality rates within 5 days of infection. In each experiment, random groups of more than 10 larvae weighing 220–280 mg were used after a 24 h fasting period. All experiments were repeated on a second experimental day for which larvae from a different batch were used. Larvae were infected by injecting 10 µl of the bacterial inoculum (~ 7 × 10^8 CFU/mL) into one of the last prolegs using a 50 µl Hamilton syringe (Merck, Darmstadt, Germany). One hour after infection, a single dose of the antibiotics was administered into the previously unused last proleg to minimize hemolymph leakage.

Preliminary experiments with MSSA (ATCC 29213) were conducted at various doses ranging from very low, subtherapeutic to human doses for both agents (1.5, 3, 6, 15, 20, 30, 50, 85, or 100 mg/kg FLX or 0.2 mg/kg FOF).The doses used for synergy testing were 0.2 mg/kg FOF, 85 mg/kg FLX or 0.2 mg/kg FOF plus 85 mg/kg FLX for the MSSA (ATCC 29213), 0.2 mg/kg FOF, 25 mg/kg FLX or 0.2 mg/kg FOF plus 25 mg/kg FLX for the second MSSA (ATCC 6538) and 0.8 mg/kg FOF, 85 mg/kg FLX or 0.8 mg/kg FOF plus 85 mg/kg FLX for the MRSA isolate (ATCC 33592). These dosages were chosen because the resulting survival rates in the monotherapy groups made it possible to demonstrate both a potential antagonistic and a synergistic effect. For FLX, a dose of 85 mg/kg commonly used in humans was used, corresponding to a dose of 2 g q8 h. This was also supported by the observation of a non-dose-dependent efficacy and even a lower survival rate when higher doses were used (Supplementary Fig. S5).

After infection, all larvae were incubated at 37 °C for the entire experimental period of 5 days, and survival was measured every 12 h. Data from these experiments were pooled and survival curves were plotted using GraphPad Prism v6.01 (GraphPad Software Inc., San Diego) and statistically compared using a log-rank test. Both infected as well as uninfected larvae, which only received sterile PBS, served as controls. In addition, potential drug toxicity of the antibiotics used was investigated by following the survival of 10 larvae each after a single dose of 200 mg/kg FOF and 200 mg/kg FLX.

For determination of in vivo bacterial loads larvae from each group receiving either 0.2, 100 or 200 mg/kg FOF, 1.5 or 85 mg/kg FLX or 0.2/1.5, 0.2/85, 100/85 or 200/85 mg/kg FOF plus FLX were randomly selected 24 h after infection and placed on ice for 1–2 min. After disinfection with 70% ethanol, hemolymph was drained directly into 1 mL Eppendorf tubes by puncturing the flank between the penultimate and last proleg with a sterile scalpel. Samples were then immediately placed on ice, and bacterial counts were determined by serial dilution and plating of 50 µL per dilution, in less than 15 min. Controls were obtained of treatment naive larvae 20 min and 24 h after infection. Individual and mean bacterial counts were plotted and statistically compared with GraphPadPrism v6.01 (GraphPad Software Inc., San Diego) using the Kruskall-Wallis test and Dunn's correction for multiple testing. DNA Extraction and Sequencing (see supplement).

### Expression of bacterial virulence factors

Relative gene expression of the most prominent virulence factors in *S. aureus* (Supplementary Table S1), namely leucotoxin (*lukDE*), accessory gene regulator A (*agrA*) and alpha-hemolysin (*hla*), was determined for methicillin-susceptible (ATCC-29213) *S. aureus employing real-time quantitative PCR (qPCR).* The housekeeping gene used was *gap*, which encodes for glyceraldehyde-3-phosphate dehydrogenase. Bacteria exposed to different antibiotic concentrations were incubated for 4 and 8 h in TSB medium (Thermo Scientific Oxoid, Austria) with 1/8, 1/4, 1/2 and 5 times the MIC for FOF, FLX or FOF plus FLX, while controls were incubated in TSB without antibiotics. For preparation of bacterial inocula, single colonies were transferred from a Columbia agar plate to fresh TSB medium and incubated overnight on an orbital shaker at 36–37 °C. This bacterial solution was further diluted in fresh TSB, incubated at 36–37 °C for 4 h hours to achieve exponential growth, then further diluted in TSB with or without antibiotics and incubated at 36 °C (± 1 °C) for 4 or 8 h. Incubated bacteria were centrifuged (18,000 g for 2 min at 4 °C), washed with ice-cold PBS and again resuspended in PBS. RNA was mechanically extracted using lysing matrix tubes (MP Biomedicals, Germany) and purified using the FavorPrep-Tissue Total RNA Mini-Kit (Favorgen Biotech Corp, Taiwan). Copy-DNA was obtained with the Onescript cDNA Synthesis-Kit (ABMgood, Canada) and used with low-ROX BrightGreend qPCR Mastermix (ABMgood, Canada) and newly designed primers (Supplementary Table S2) on a Quantstudio 3 (ThermoFisher Scientific, Austria). All samples were analyzed in quadruplicates, relative gene expression values were calculated by ΔΔCt using no treatment controls as references and are stated as mean relative gene expression values ± SD (RQ ± SD). Bioinformatics analysis (see supplement).

## Results

### Antimicrobial susceptibility and synergy testing

In Supplementary Table S3 the average and median MIC values in mg/L of FLX with and without G6P are shown. No difference in the evaluated MIC value could be detected between the two approaches. In Supplementary Table S4 the individual average MIC values with FOF and FLX for all strains with standard deviation are depicted.

The median individual MIC values of FOF and FLX, the median combined MICs of FOF/FLX, the FICI indicating an overall synergistic or additive effect of FOF and FLX and the SBPI, are shown in Table 1.

**Table 1:** The median minimum inhibitory concentrations (MIC) of fosfomycin (FOF) and flucloxacillin (FLX) alone and in combination are shown

Isolates	MIC in mg/L		FICI- interpretation^a^ (mean-FICI ± SD)	Combined MICs (FOF; FLX)^b^	Recovered FLX susceptibility (min combined FOF-MIC)^c^	SBPI^d^
	FOF	FLX				
MSSA						
ATCC-29213	2	0.5	AE (0.71 ± 0.4)	1/4; 1/4	n.a	72
MSSA 231/20	4	0.125	NI (1.15 ± 0.54)	1/2; 1/4	n.a	49
MSSA 280/20	4	0.25	AE (0.83 ± 0.26)	1/4; 1/4	n.a	49
MSSA 249/20	1	0.25	AE (0.69 ± 0.24)	1/2; 1/4	n.a	86
MSSA 204/20	4	0.125	AE (0.66 ± 0.45)	1/4; 1/4	n.a	65
MRSA			0.25, 0.125, 0.06, 0.03, 0.015			
ATCC 33592 (FOF S)	8	8	Sy (0.21 ± 0.06)	1/8; 1/8	S (≥ 1/8)	33
23,622 DSMZ (FOF R)	128	1024	Sy (0.33 ± 0.29)	1/8; 1/64	R	2
MRSA 874/19	16	1	Sy (0.31 ± 0.12)	1/4; 1/8	S (≥ 1/8)	13
MRSA 845/19	0.5	0.5	Sy (0.48 ± 0.11)	1/2; 1/8	n.a	139
MRSA 563/18	2	0.5	AE (0.69 ± 0.24)	1/4; 1/8	n.a	75

*FICI* fractional inhibitory concentration index, *SBPI* susceptible breakpoint index, *MSSA* methicillin-susceptible *S. aureus*, *MRSA* methicillin-resistant *S. aureus*, *ATCC* American type culture collection, *DSMZ* german collection of microorganisms and cell cultures

^a^Interpretation of the FICI: Sy, synergism =  ≤ 0.5, AE, additive effect = 0.5– ≤ 1.0, NI, no interaction =  > 1 and < 4; An, antagonism =  ≥ 4, followed by the mean FICI ± SD in brackets

^b^Combined minimum inhibitory concentrations of flucloxacillin and fosfomycin used for calculation of the fractional inhibitory concentration index stated as relative concentrations of their respective MICs

^c^Stated as susceptible (S). Stated as resistant (R). Numbers in brackets: the lowest fosfomycin concentrations which resulted in susceptible flucloxacillin MICs are stated as times of their MIC. N. a. not applicable due to the flucloxacillin MIC below the PK/PD breakpoint

^d^Susceptible Breakpoint Index (SBPI). An SBPI ≥ 2 indicates that the combined MICs of the tested antimicrobials are equally or lower than their respective breakpoints. The greater the SBPI value, the more effective the antimicrobial combination. All experiments were performed up to 10 times and at least 5 times

FOF and FLX in combination could lower the MIC up to 64-fold in tests with MRSA isolates and up to fourfold with MSSA isolates (e.g., isolate DSMZ-23622 from 1024 mg/L FLX to 16 mg/L in combination with 16 mg/L FOF and strain MSSA-249/20 from 0.25 mg/L FLX to 0.063 mg/L in combination with 0.5 mg/L FOF). Moreover, the SBPI ≥ 2 indicates that the combined MICs of the tested antimicrobials are equally or lower than their respective breakpoints (Table 1).

### Time kill curves

**Fig. 1:**
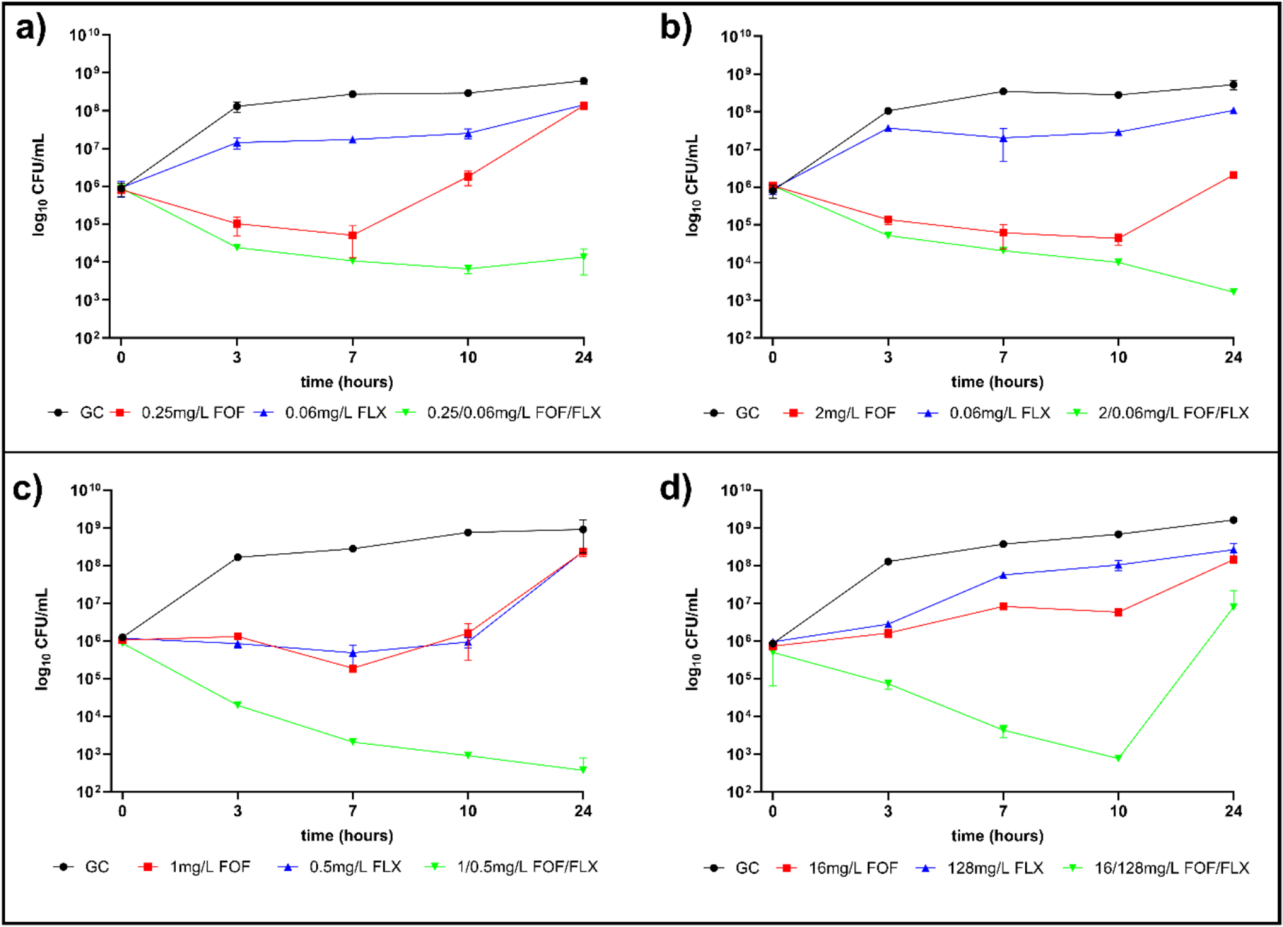
CFU/mL with standard deviation (partly overlaid by symbols) of the Time Kill Curves with fosfomycin (FOF) and flucloxacillin (FLX) of two MSSA strains **a** ATCC 29213 and **b** MSSA 231/20 and two MRSA strains **c** ATCC 33592 (FOF susceptible) and **d** DSMZ 23622 (FOF resistant) are shown over 24 h. The individual MICs of the strains are **a** FOF 2 mg/L and FLX 0.5 mg/L, **b** FOF 4 mg/L and FLX 0.125 mg/L, **c** FOF 8 mg/L, FLX 8 mg/L and **d** FOF 128 mg/L, FLX 1024 mg/L. Concentrations evaluated by the checkerboard assay showing synergy or additive behavior were tested in single and in combination. Circles represent the growth control, red and blue symbols depict individual concentrations of FOF and FLX respectively and green symbols show the combination of both antibiotics

TKC with two MSSA strains (ATCC-29213 and MSSA 231/20) Fig. 1a and b and two MRSA strains (23622 DSMZ (FOF R) and ATCC 33592 (FOF S)) Fig. 1 c and d with concentrations evaluated by the checkerboard assay showing synergy or additive behavior were tested in single and in combination. In Fig. 1 only excerpts of these data sets are depicted. All other TKC with further concentrations tested are depicted in Supplementary Figs. S1–S4. Overall, concentrations tested in combination did show beneficial effects on killing in every approach compared to single administrations and after 24 h a reduction of at least 2 log10 CFU/mL was observed for most of the combinations and regrowth was present in only few cases of combined FOF and FLX approaches.

In Fig. 1a and b TKC with MSSA strains ATCC 29213 and MSSA 231/20 show that 1 log10 killing could be achieved when FOF was administered alone (1/8 MIC and ½ MIC, respectively) but after 24 h regrowth to the initial inoculum and above occurred. With FLX alone (1/8 MIC and ½ MIC, respectively) growth was impaired. Contrary, the combination of the individual FOF and FLX concentrations achieved a 2 log 10 killing for both strains without regrowth.

In Fig. 1 c and d TKC with MRSA strains ATCC 33592(FOF S) and DSMZ 23622 (FOF R) show that only the combination of FOF and FLX could achieve a killing of 3 log10 for both strains up to 10 h. While the FOF-susceptible strain was further inhibited in its growth until the 24 h time point the FOF-resistant strain showed regrowth and reached again the initial inoculum.

### In vivo* G. mellonella survival assay*

Preliminary *G. mellonella* survival experiments after infection with MSSA (ATCC 29213) are shown in Supplementary Fig. S5. In summary, regardless of the adjusted mortality in the control group and the FLX dose used (FLX 1.5, 3, 6, 15, 20, 30, 50, 85, 100 mg/kg), therapy with FLX showed similar or worse results than the untreated control, whereas FOF improved the survival even at very low, subtherapeutic doses.

**Fig. 2:**
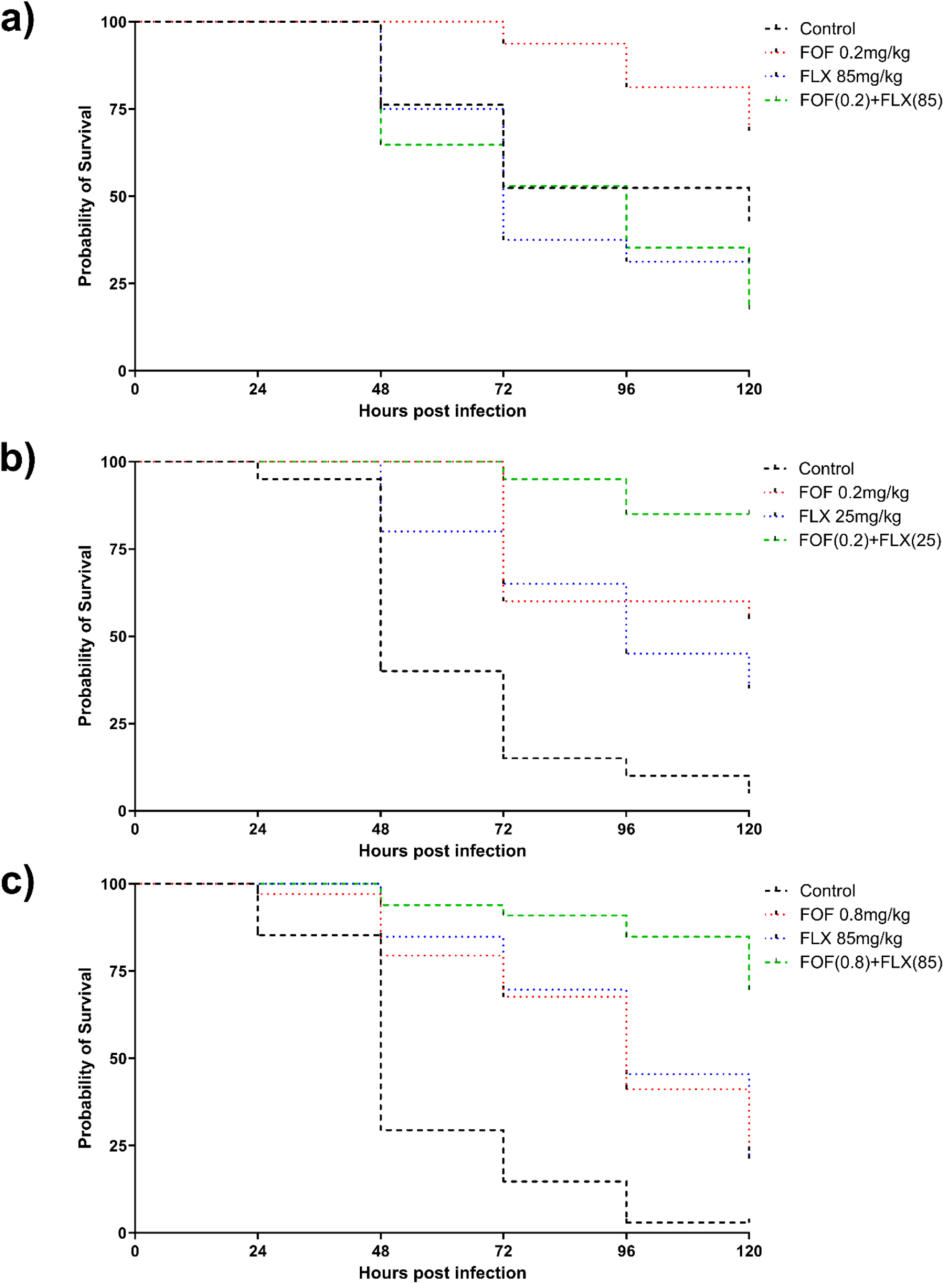
**a** Survival curves of *G. mellonella* larvae infected with methicillin-susceptible *S. aureus* (ATCC 29213) followed by treatment with flucloxacillin (FLX) 85 mg/kg (n = 16), fosfomycin (FOF) 0.2 mg/kg (n = 16) or the combination of both, FLX 85 mg/kg plus FOF 0.2 mg/kg (n = 17). Larvae in the control group were infected but received only sterile PBS (n = 21). **b** Survival curves of *G. mellonella* larvae infected with methicillin-susceptible *S. aureus* (ATCC 6538) followed by treatment with FLX 85 mg/kg (n = 20), FOF 0.2 mg/kg (n = 20) or the combination of both, FLX 85 mg/kg plus FOF 0.2 mg/kg (n = 20). Larvae in the control group were infected but received only sterile PBS (n = 20). **c** Survival curves of *G. mellonella* larvae infected with methicillin-resistant *S. aureus* (ATCC 33592) followed by treatment with FLX 85 mg/kg (n = 33), FOF 0.8 mg/kg (n = 34) or the combination of both, FLX 85 mg/kg plus FOF 0.8 mg/kg (n = 33). Larvae in the control group were infected but received only sterile PBS (n = 34)

In the main experiment, infection with the MSSA isolate (ATCC 29213) resulted in a survival rate of 43% in the control group (n = 9/21). Treatment with low-dose FOF-alone (0.2 mg/kg) increased survival to 69% (n = 11/16), whereas survival decreased to 31% (n = 5/16) and 18% (n = 3/17) in the FLX-alone (85 mg/kg) and the combination treatment group with FLX plus low-dose FOF, respectively (Fig. 2a).

In vivo bacterial counts determined at baseline and 24 h postinfection with MSSA ATCC 29213 during therapy with FOF (0.2, 100, and 200 mg/kg), FLX (1.5 and 85 mg/kg), and their combinations are shown in Supplementary Fig. S6. With the exception of therapy with low-dose FLX (1.5 mg/kg), which showed a significantly higher bacterial count after 24 h, no differences were observed between treatment groups in any experiment.

Toxicity tests with FLX alone (200 mg/kg), FOF alone (200 mg/kg) and FLX (200 mg/kg) plus FOF (200 mg/kg) showed no adverse effects with 100% survival in each group (data not shown).

The experiments with the alternative MSSA isolate (ATCC 6538) resulted in a survival rate of 5% (n = 1/20) in the control group while the monotherapy groups receiving low-dose FOF and FLX had a survival rate of 55% (n = 11/20) and 35% (n = 7/20) respectively. The combination therapy with FOF plus FLX led to a significantly better survival rate of 85% (n = 17/20) (p < 0.0001) (Fig. 2b).

After infection with the MRSA isolate (ATCC 33592), the survival rate in the control group was 3% (n = 1/34). The treatment groups with FLX alone (85 mg/kg) and low-dose FOF alone (0.8 mg/kg) had similar survival rates of 21% (n = 7/33) and 24% (n = 8/34), respectively, while the combination treatment group with FLX (85 mg/kg) plus low-dose FOF (0.8 mg/kg) showed a significantly better survival rate of 70% (n = 23/33) compared to the monotherapies (p < 0.0001) (Fig. 2c). DNA Extraction and Sequencing (see supplement).

### Expression of bacterial virulence factors

**Fig. 3:**
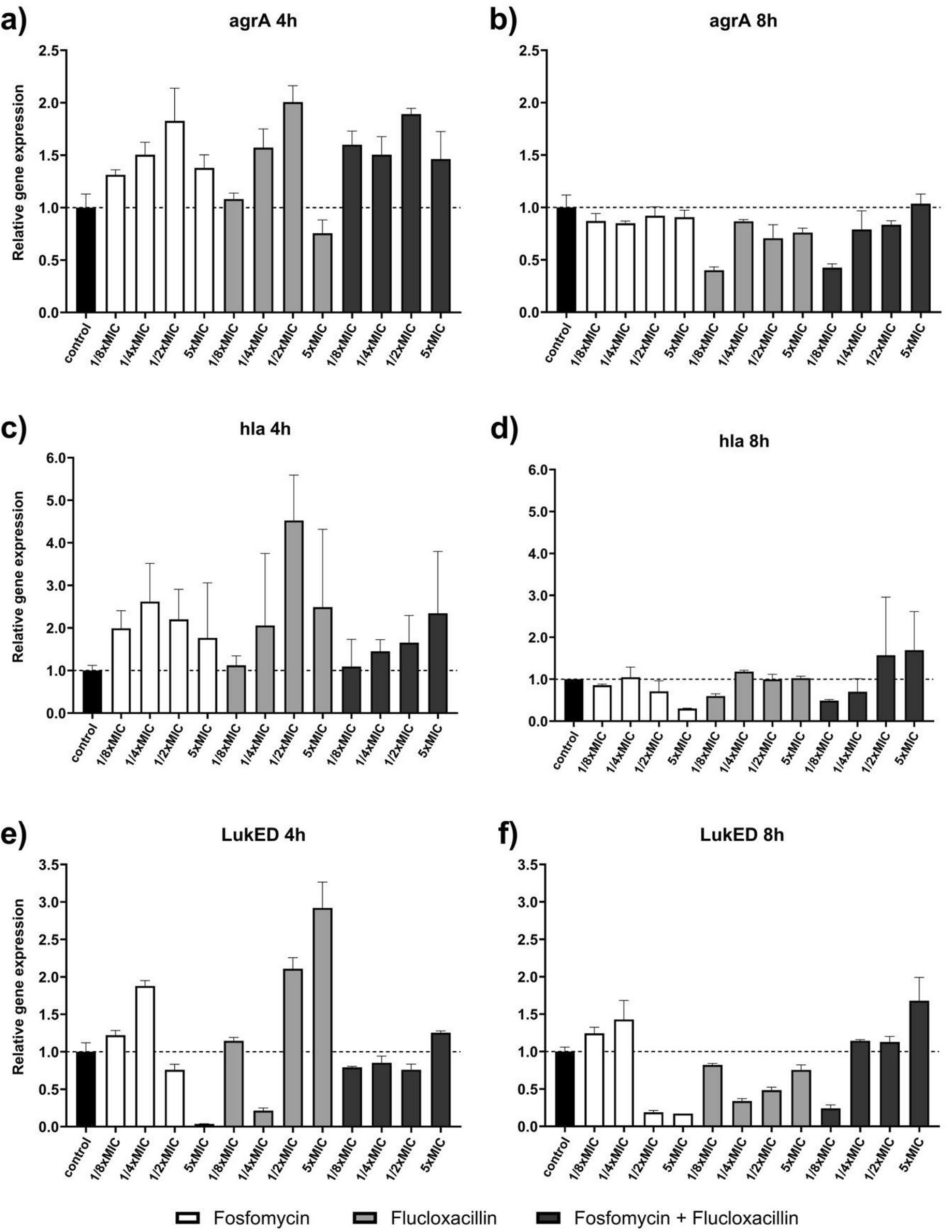
**a-f** Relative gene expression of selected virulence factors/gene regulators (accessory gene regulator A (*agrA*), alpha-hemolysin (*hla*), leukotoxin ED (*lukED*)) of methicillin-susceptible *S. aureus* ATCC 29213 determined by RT-PCR with glyceraldehyde 3-phosphate dehydrogenase (*gap*) as housekeeping gene and an untreated control as reference. Bacteria were exposed to either fosfomycin or flucloxacillin at concentrations equivalent to 1/8, ¼, ½ or 5 times the respective minimum inhibitory concentration (MIC) for a period of 4 or 8 h, respectively. Data are expressed as mean (± SD) relative quantification values

Given the surprising outcomes observed for MSSA and FLX monotherapy in the *G. mellonella* experiments, conducting an expression analysis of virulence factors seemed warranted. The relative gene expression of *agrA*, *hla*, and *lukDE* is shown in Fig. 3. Gene *agrA* showed a slight decrease in expression after an 8-h exposure to 1/8 times the MIC of FLU (RQ 0.40 ± 0.03) or the combination of FOF + FLU (RQ 0.43 ± 0.04). After 4-h exposure, *hla* showed increased expression for all antibiotics and concentrations tested with ½-fold MIC of FLX showing the highest increase (RQ 4.53 ± 1.07) and the remaining values ranging from 1.1 to 2.6. Prolonged exposure to therapeutic concentrations of fosfomycin (5xMIC, RQ 0.30 ± 0.01) or subinhibitory concentrations of FLU (1/8xMIC, RQ 0.59 ± 0.06) or FOF plus FLU (1/8xMIC, RQ 0.49 ± 0.03) resulted in reduced *hla* expression. The most pronounced changes were observed with *lukED*. While FOF showed significantly decreased expression after 4 h exposure to 5xMIC (RQ 0.04 ± 0.00) and after 8 h exposure to 1/2xMIC (RQ 0.19 ± 0.03) and 5xMIC (RQ 0.17 ± 0.00), FLU increased gene expression after 4 h exposure to 1/2xMIC (RQ 2.11 ± 0.15) and 5xMIC (RQ 2.92 ± 0.34).

## Discussion

FOF, known for its broad-spectrum antimicrobial activity, particularly against *S. aureus* and methicillin-resistant staphylococci, is often explored in combination therapies to prevent the rapid emergence of resistant mutants and to sterilize the infection more rapidly [17, 18]. Notably, previous studies have shown promising synergistic effects with various beta-lactam and non-beta lactam antimicrobials, including cefpirome, cefazolin, cefotaxim, oxacillin, and imipenem [19–22].

The present study aimed to assess the synergistic effects of FLX and FOF against various *S.s aureus* strains, including methicillin-resistant isolates. Until now, only a limited number of studies have adopted a comprehensive multidimensional approach similar to ours, which integrates antimicrobial susceptibility testing, synergy testing, TKC’s, in vivo *G. mellonella* survival assays, and gene expression analyses of bacterial virulence factors.

The synergy testing revealed that the combination of FLX and FOF significantly lowered the MIC, particularly in methicillin-resistant strains ranging from 1/8 to 1/64 of their MIC. The susceptible breakpoint index (SBPI) values indicated that the combined MICs were equal to or lower than their respective breakpoints, as the greater the SBPI value (≥ 2), the more effective the antimicrobial combination (e.g., SBPI for MSSA 49–86 and for MRSA 2–139). Results of TKCs further supported this synergistic potential, demonstrating not only inhibition of bacterial growth but also prevention of regrowth, indicating sustained effectiveness. In both methicillin-susceptible and methicillin-resistant strains, the combination exhibited superior bacterial killing compared to individual antibiotic treatments. TKC data provided robust evidence for the relevance of the FOF and FLX combination, emphasizing its potential to enhance antibiotic efficacy. Although TKCs with MSSA strains ATCC 29213 and MSSA 231/20 exhibited a 1 log10 reduction in bacterial count with FOF alone, regrowth occurred after 24 h. FLX alone effectively inhibited growth, but when used in combination, it achieved a 2 log10 reduction in bacterial count for both strains without any subsequent regrowth. For MRSA strains 23622 DSMZ (FOF R) and ATCC 33592 (FOF S), only the combination of FOF and FLX achieved a 3 log10 reduction in bacterial count for both strains within the first 10 h. The FOF S strain demonstrated sustained growth inhibition up to 24 h, whereas the FOF R strain showed regrowth. Consequently, this data highlights significant potential against both MSSA and MRSA isolates. To further advance our research, we conducted in vivo testing to evaluate the potential combination in larvae. The decision to utilize the *G. mellonella* model for in vivo testing was informed by its established efficacy in simulating bacterial infections and assessing antimicrobial efficacy as discussed by Serrano et al. [23]. This model, first researched over 85 years ago, serves as a valuable tool for studying infections caused by various pathogens, screening antimicrobials, and exploring immune responses. Notably, its immune system exhibits similarities with mammals, and outcomes often align with mammalian and other invertebrate models [24]. Despite inherent limitations, *G. mellonella* effectively bridges the gap between in vitro and mammalian in vivo studies, reflecting the principles of the 3Rs in animal experimentation.

The *G. mellonella* survival assays offered insights into the in vivo efficacy of the FOF and FLX combination. The *G. mellonella* model provided a valuable platform for assessing the therapeutic advantage of the combination, with particularly promising results in MRSA infections. In MRSA infection, even the combination of FLX plus low-dose FOF significantly improved survival rates compared to monotherapies. These findings suggest that the FOF and FLX combination could be a viable option for treating *S. aureu*s infections, including those caused by drug-resistant strains. For MSSA infections, however, this could not be proven at the beginning, as treatment with FLX or FLX plus low-dose FOF after infection with MSSA (ATCC 29213) surprisingly led to poorer survival than low-dose FOF alone and even placebo. In order to better understand this observation, further in vivo experiments were carried out with FLX alone, FOF alone, and the combination of both at different doses, in which the bacterial counts in the hemolymph were determined before administration of the therapy and after 24 h. In contrast to the results of the survival assays, however, there were no differences between the treatment groups. The complexity of regulatory circuits in *S. aureus* contributes to its adaptability and virulence, with genes encoding toxins (e.g., *lukED, hla*), cell surface proteins (e.g., protein A), and antimicrobial resistance (e.g., *agrA*) [25, 26]. Given the surprising results of the *G. mellonella* survival assay for infection with MSSA and FLX therapy, an expression analysis was added. The virulence factors of ATCC 29213 were determined using whole genome sequencing, and then *agrA*, a major global regulator of *S. aureus* virulence and two factors (*hla, lukDE*) that have already shown an effect on mortality in animal experiments after gene knockout or expression changes were examined. The gene expression analyses shed light on the impact of FOF and FLX alone and in combination at different concentrations on the of bacterial virulence factors. As suspected, monotherapy of FLX enhanced the gene expression of all virulence factors tested after 4 h, with *lukED* being the most pronounced [27–29]. In contrast, only the expression of *lukED* was significantly reduced after exposure to FOF alone, while the combination of FOF and FLX showed a lower reduction of *lukED*, but also of *agrA* and *hla* after exposure to 1/8 of the MIC. The lower influence on the expression of virulence factors under combination therapy with FOF plus FLX compared to FOF alone could therefore also explain the significantly reduced survival in the *G. mellonella* survival assay after infection with MSSA ATCC 29213.

However, MSSA ATCC 29213 is only one of many reference strains and it was unclear whether these results are transferable to other MSSA strains. For this reason, further in vivo experiments were carried out with another MSSA isolate (ATCC 6538). In a preliminary experiment, treatment with FLX at a dose of 100 mg/kg resulted in a survival rate of 91% compared to 0% in the control group, which was consistent with the previously proposed hypothesis (data not shown). In addition, survival rates of 5% in the control group, 55% in the group receiving low-dose FOF only, 35% in the group receiving FLX only, and 85% in the group receiving a combination of low-dose FOF and FLX were observed in the main experiment, similar to the studies with the MRSA isolate.

Based on the data obtained in this study and the current literature, the combination of FOF plus FLX could be prospectively investigated in clinical trials with specific areas of application. On the one hand, it might be investigated instead of imipenem plus FOF as a rescue therapy after treatment failure or necessary discontinuation due to pronounced side effects of classic MRSA agents, thereby ensuring that carbapenems are retained as last resort antibiotics for the treatment of critically ill patients. On the other hand, this combination therapy could be used especially in countries with low to moderate MRSA rates for the initial, calculated antimicrobial therapy of suspected *S. aureus* infections, such as delayed prosthetic joint infections or early prosthetic infective endocarditis, as well as in cases where *S. aureus* has been detected as the causative pathogen but the results of an antimicrobial susceptibility test are still pending or missing, in order to utilize the advantages of such combination therapy in the initial treatment phase.

In conclusion, this study provides substantial evidence supporting the synergistic effects of FLX and FOF against *S. aureus*, particularly in drug-resistant strains. The multidimensional approach, encompassing MIC assessments, TKC, in vivo assays, and gene expression analyses, provides a comprehensive understanding of the combination's potential. Nevertheless, it is important to acknowledge that findings from translational models, such as *G. mellonella*, may not always be directly extrapolatable to clinical settings. Differences in immune response, pharmacokinetics, and host–pathogen interactions highlight the limitations of preclinical models and underscore the need for further validation in human studies.

## Supplementary Information

Below is the link to the electronic supplementary material.

Supplementary file1 (DOCX 1724 KB)

## Data Availability

The NGS data of S. aureus ATCC 29213 have been deposited in the NCBI genome database (BioProject PRJNA1063577).
