# Supplementary material for: Synergistic activity of fosfomycin and flucloxacillin against methicillin-susceptible and methicillin-resistant Staphylococcus aureus: in vitro and in vivo assessment
Source: Med Microbiol Immunol. 2025 Jun 21;214(1):32. doi: 10.1007/s00430-025-00841-3 (PMC12182452; doi:10.1007/s00430-025-00841-3)
Supplement: Supplementary file 1 — Supplementary file1 (DOCX 1724 KB) [file 430_2025_841_MOESM1_ESM.docx]

**Supplementary methods:**

**Table S1:** *Staphylococcus aureus* ATCC 29213 virulence factors determined by whole genome sequencing.

| **Staphylococcus aureus ATCC 29213 virulence factors** |  |
| --- | --- |
| Staphylococcal enterotoxin A | Sea |
|  |  |
| Staphylococcal enterotoxin G | Seg |
|  |  |
| Staphylococcal enterotoxin I | Sei |
|  |  |
| Staphylococcal enterotoxin M | Sem |
|  |  |
| Staphylococcal enterotoxin N | Sen |
|  |  |
| Staphylococcal enterotoxin O | Seo |
|  |  |
| Staphylococcal enterotoxin-like X | Selx |
|  |  |
| Clumping factor A | clfA |
|  |  |
| Clumping factor B | clfB |
|  |  |
| Fibronectin-binding protein A | fnbA |
|  |  |
| Fibrinogen-binding protein | Fib |
|  |  |
| LukE-LukD | LukED |
|  |  |
| Alpha hemolysin | Hla |
|  |  |
| Delta-hemolysin | Hld |
|  |  |
| Gamma-hemolysin variant | Hlg-2 |
|  |  |

**Table S2:** Oligonucleotides used to RT-PCR

## ***DNA Extraction and Sequencing***

DNA extraction was performed using a modified phenol-chloroform method. Briefly, bacterial colonies incubated overnight were transferred to 1.5 mL tubes with 400 µL phenol-chloroform-isoamylalcohol (25:24:1). The suspensions were then transferred to

|  | **Name** | ***Gene*** | **Primer-name** | **Sequence (5‘-3‘)** | **Length (bp)** |
| --- | --- | --- | --- | --- | --- |
| **Virulence genes** | Leucotoxin | *lukED* | LukED-fwd_806 | ATGCGACTTTATTCCCTAGAAC | 236 |
|  |  |  | LukED-rev_1041 | CTCGCTTACAGGTGTGATATG |  |
|  |  |  |  |  |  |
|  | Gamma-hemolysin A,B,C | *hlg* | hlg-fwd_470 | ACTTCCAATCAGCGCCATC | 132 |
|  |  |  | hlg-fwd_601 | CTTTCACTCCCCATTTAACACC |  |
|  |  |  |  |  |  |
|  | Accessory gene regulator A | *agrA* | agrA-fwd_100 | GCCCTCGCAACTGATAATCC | 216 |
|  |  |  | agrA-rev_315 | CATCGCTGCAACTTTGTAGAC |  |
|  |  |  |  |  |  |
|  | Alpha-hemolysin | *hla* | HLA-1 | CTGATTACTATCCAAGAAATTCGATTG | 209 |
|  |  |  | HLA-2 | CTTTCCAGCCTACTTTTTTATCAGT |  |
|  |  |  |  |  |  |
| **Housekeeping gene** | Glyceraldehyde 3-phosphate dehydrogenase | *gap* | gap-f | CAAAATACACAAGACGCACC | 103 |
|  |  |  | gap1-r | CCGATAGCTTTAGCAGCAC |  |
|  |  |  |  |  |  |

lysing tubes (Lysing Matrix E 2 mL Tube, MP Biomedicals Germany GmbH, Eschwege, Germany) and homogenised using the Precellys® 24 (PEQLAB Biotechnologies GmbH, Polling, Austria).

After centrifugation (5min, 18 000 g) of the supernatant, the upper aqueous phase was transferred to a new tube containing chloroform (300µL chloroform) and centrifuged again.

After repeating this step, the upper aqueous phase was added to a new tube containing 1M-ammonium acetate (225 µL) to assist precipitation. The suspension was shaken for 5 seconds and then made up to 1.8 mL with ice-cold 100 % ethanol and mixed by inverting. After 30 min incubation on ice and centrifugation (15 min, 18 000 g, 4°C), the supernatant was discarded, and the DNA pellet was washed twice with 70 % ethanol. Purity was measured with Nanodrop One and concentrations were determined with the Qubit 4.0 Fluorometer (both Thermo Fisher Scientific, Waltham, Massachusetts) using the Qubit dsDNA BR Assay Kit. An absorbance ratio 260/280 of >1.8 was assumed to be a pure, protein-free sample, and a ratio 260/230 of >2.0 was assumed to be also free of other contaminants such as chloroform or free nucleotides. DNA library preparation was performed according to manufacturer’s instructions using the Nextera DNA Library Preparation Kit (Illumina, San Diego, California). Sequencing was perfomed on the MiSeq platform using a V3 Flowcell (both Illumina, San Diego, California).

## ***Bioinformatics analysis***

Trim Galore v0.6.5 was used to remove lower quality bases and ensure a read length of at least 90 bp [1]. SPAdes v3.15.2 was used for assembling and QUAST for quality testing [2,3]. For read mapping and variant calling Bowtie2 v2.4.2 and VarScan v2.4.4 were used, respectively [4,5]. Structural analysis of the assemblies and larger INDELs were performed using Mauve. (https://www.ncbi.nlm.nih.gov/pmc/articles/PMC442156/)

**Supplementary Results**

**Table S3:**

|  | **MIC FLX with G6P in mg/L (triplicates)** | | | **MIC FLX without G6P in mg/L (triplicates)** | | |
| --- | --- | --- | --- | --- | --- | --- |
| **Isolates:** | **Average** | **SD** | **Median** | **Average** | **SD** | **Median** |
| **MSSA** |  |  |  |  |  |  |
| ATCC-29213 | 0.58 | 0.20 | 0.5 | 0.58 | 0.20 | 0.5 |
| MSSA 231/20 | 0.12 | 0 | 0.125 | 0.125 | 0.13 | 0 |
| MSSA 280/20 | 0.25 | 0 | 0.25 | 0.25 | 0 | 0.25 |
| MSSA 249/20 | 0.25 | 0 | 0.25 | 0.25 | 0 | 0.25 |
| MSSA 204/20 | 0.17 | 0.06 | 0.125 | 0.21 | 0.06 | 0.125 |
| **MRSA** |  |  |  |  |  |  |
| ATCC 33592 (fosfomycin susceptible) | 8 | 0 | 8 | 8 | 0 | 8 |
| 23622 DSMZ (fosfomycin resistant) | 1024 | 0 | 1024 | 1024 | 0 | 1024 |
| MRSA 874/19 | 1 | 0 | 1 | 1 | 0 | 1 |
| MRSA 845/19 | 0.5 | 0 | 0.5 | 0.5 | 0 | 0.5 |
| MRSA 563/18 | 0.5 | 0 | 0.5 | 0.5 | 0 | 0.5 |

**Table S3:** The average (with standard deviation (SD)) and median MIC values of flucloxacillin (FLX) are given in mg/L tested with and without Glucose-6-Phosphate(G6P) against MSSA and MRSA strains. Abbreviations: MSSA, methicillin-susceptible Staphylococcus aureus; MRSA methicillin-resistant Staphylococcus aureus; ATCC, American type culture collection; DSMZ, german collection of microorganisms and cell cultures.

**Table S4:**

|  | **FOF** | | | **FLX** | | |
| --- | --- | --- | --- | --- | --- | --- |
| **Isolates:** | **Average MIC in mg/L** | **SD** | **Median MIC in mg/L** | **Average MIC in mg/mL** | **SD** | **Median MIC in mg/L** |
| **MSSA** |  |  |  |  |  |  |
| ATCC-29213 | 1.92 | 0.75 | 2 | 0.58 | 0.20 | 0.5 |
| MSSA 231/20 | 4.4 | 1.26 | 4 | 0.125 | 0 | 0.125 |
| MSSA 280/20 | 4 | 1.63 | 4 | 0.25 | 0 | 0.25 |
| MSSA 249/20 | 1.3 | 0.47 | 1 | 0.25 | 0 | 0.25 |
| MSSA 204/20 | 3.4 | 2.1 | 4 | 0.21 | 0.06 | 0.125 |
| **MRSA** |  |  |  |  |  |  |
| ATCC 33592  (FOF S) | 12 | 4.3 | 8 | 8 | 0 | 8 |
| 23622 DSMZ  (FOF R) | 128 | 0 | 128 | 1024 | 0 | 1024 |
| MRSA 874/19 | 16 | 0 | 16 | 1 | 0 | 1 |
| MRSA 845/19 | 0.67 | 0.25 | 0.5 | 0.5 | 0 | 0.5 |
| MRSA 563/18 | 1.83 | 0.79 | 2 | 0.5 | 0 | 0.5 |

**Table S4:** The average (with standard deviation (SD)) and median MIC values of fosfomycin (FOF) and flucloxacillin (FLX) are given in mg/L. Abbreviations: MSSA, methicillin-susceptible *Staphylococcus aureus*; MRSA methicillin-resistant *Staphylococcus aureus*; ATCC, American type culture collection; DSMZ, german collection of microorganisms and cell cultures.

**Figure S1**: CFU/mL with standard deviation (partly overlaid by symbols) of the Time Kill Curves of MSSA ATCC-29213 with fosfomycin (FOF) and flucloxacillin (FLX) in single **a)** and in combination **b)** are shown over 24 hours. The individual MIC of the strain is FOF 2mg/L and FLX 0.5mg/L. **
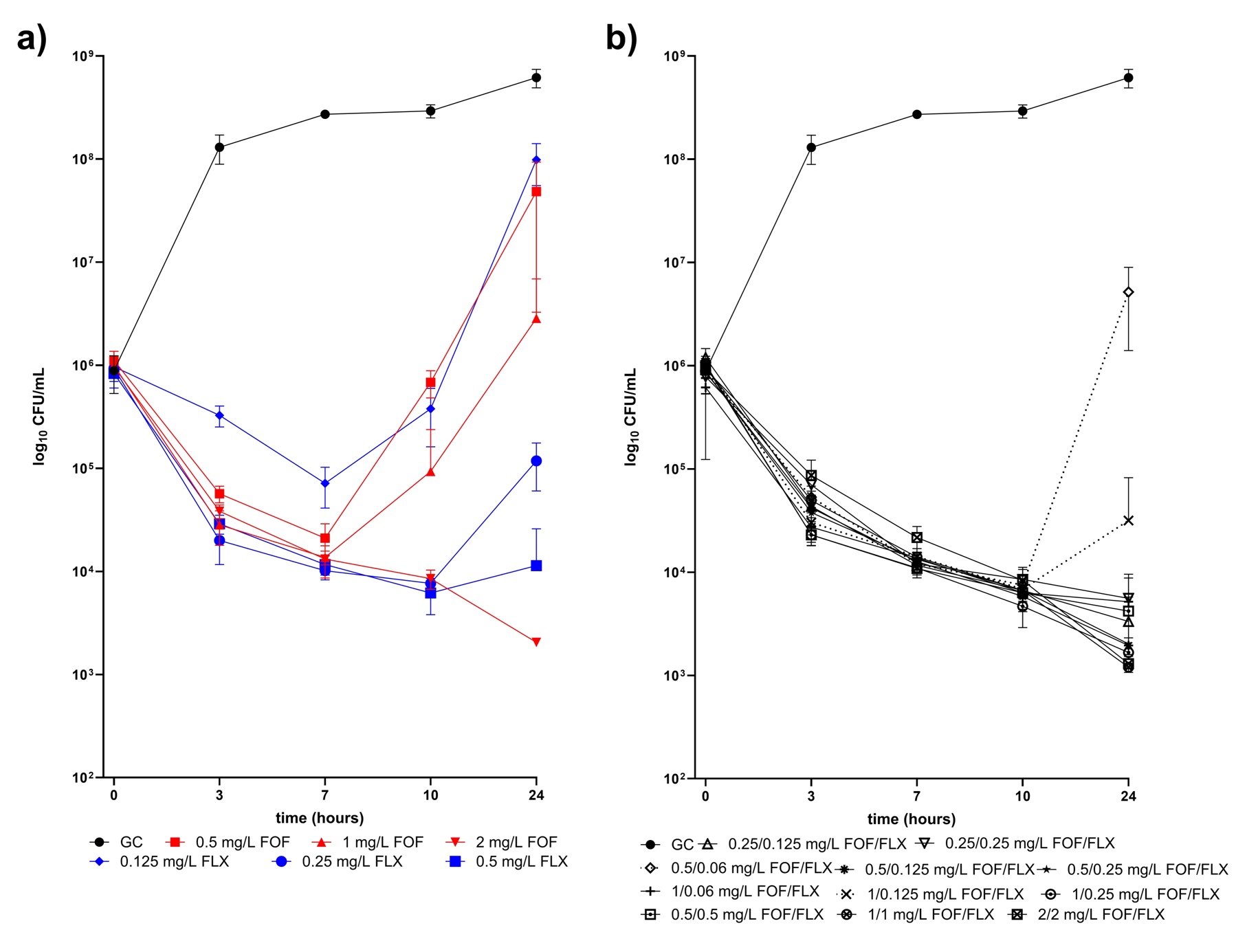
**

**Figure S2***:* CFU/mL with standard deviation (partly overlaid by symbols) of the Time Kill Curves of MSSA 231-20 with fosfomycin (FOF) and flucloxacillin (FLX) in single **a)** and in combination **b)** are shown over 24 hours. The individual MIC of the strain is FOF 4mg/L and FLX 0.125mg/
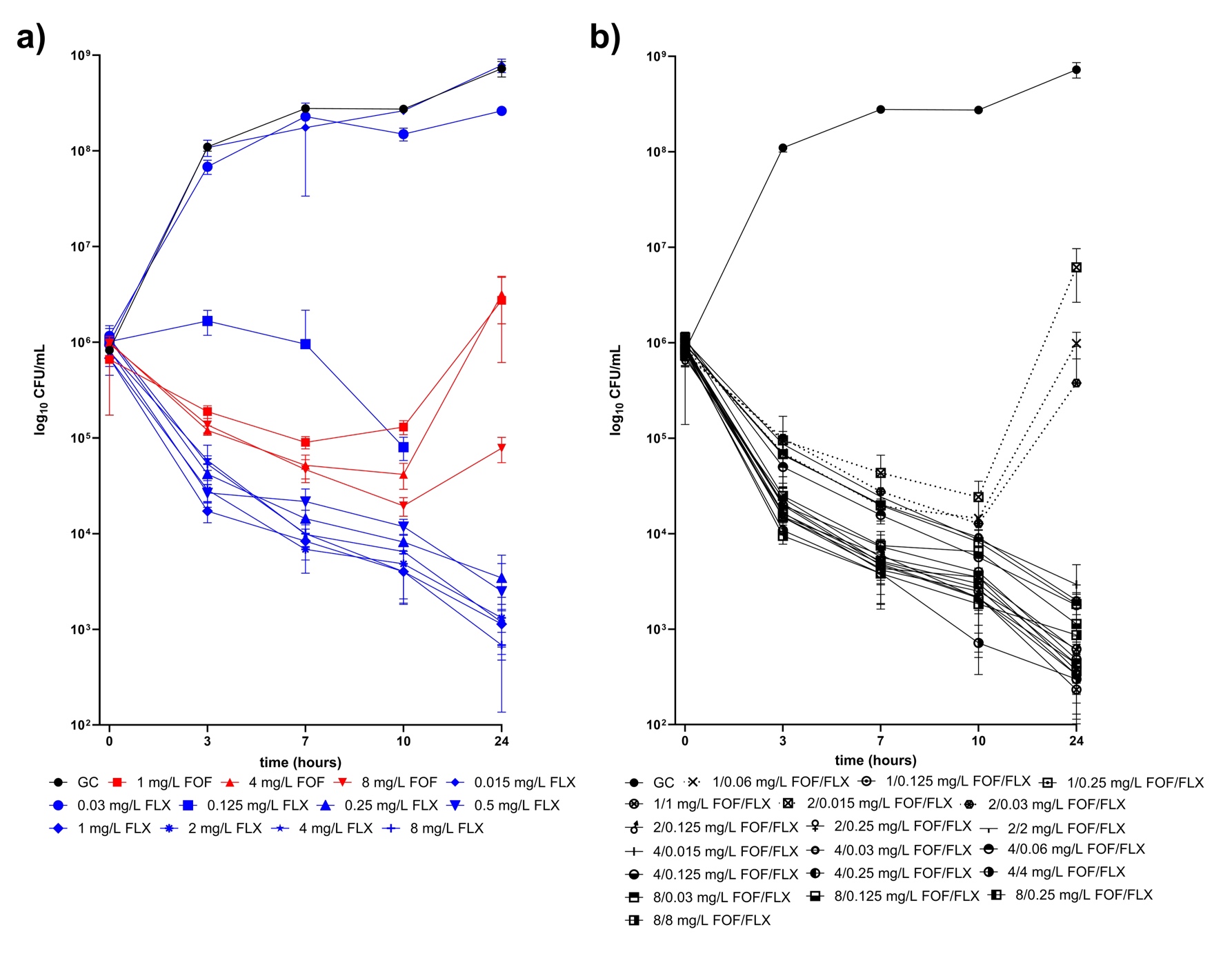


**Figure S3***:* CFU/mL with standard deviation (partly overlaid by symbols) of the Time Kill Curves of MRSA ATCC 33592 (FOF S) with fosfomycin (FOF) and flucloxacillin (FLX) in single **a)** and in combination **b)** are shown over 24 hours. The individual MIC of the strain is FOF 8mg/L and FLX 8mg/L.


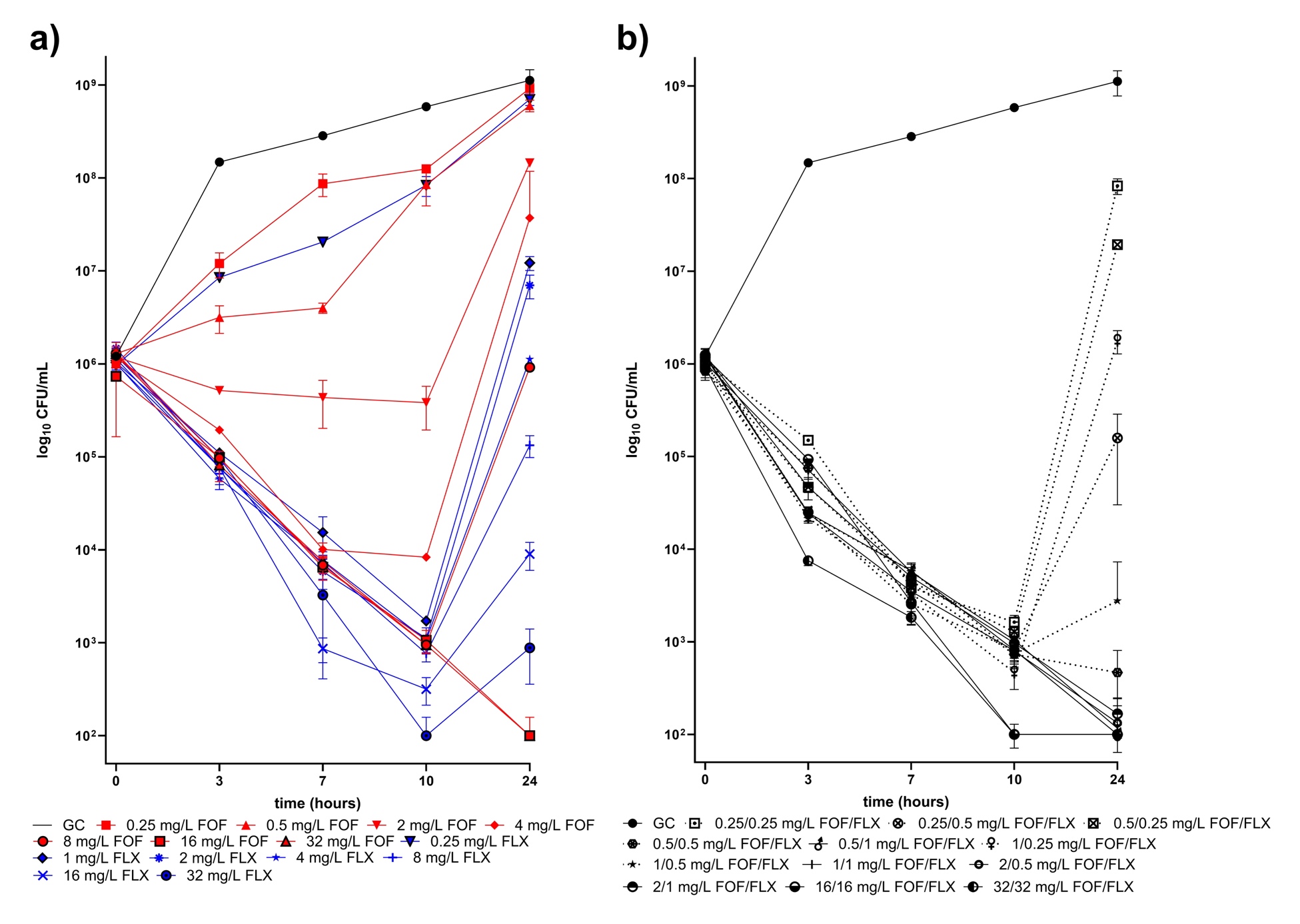


**Figure S4***:* CFU/mL with standard deviation (partly overlaid by symbols) of the Time Kill Curves of MRSA DSMZ 23622 (FOF R) with fosfomycin (FOF) and flucloxacillin (FLX) in single **a)** and in combination **b)** are shown over 24 hours. The individual MIC of the strain is FOF 128mg/L and FLX 1024mg/L.


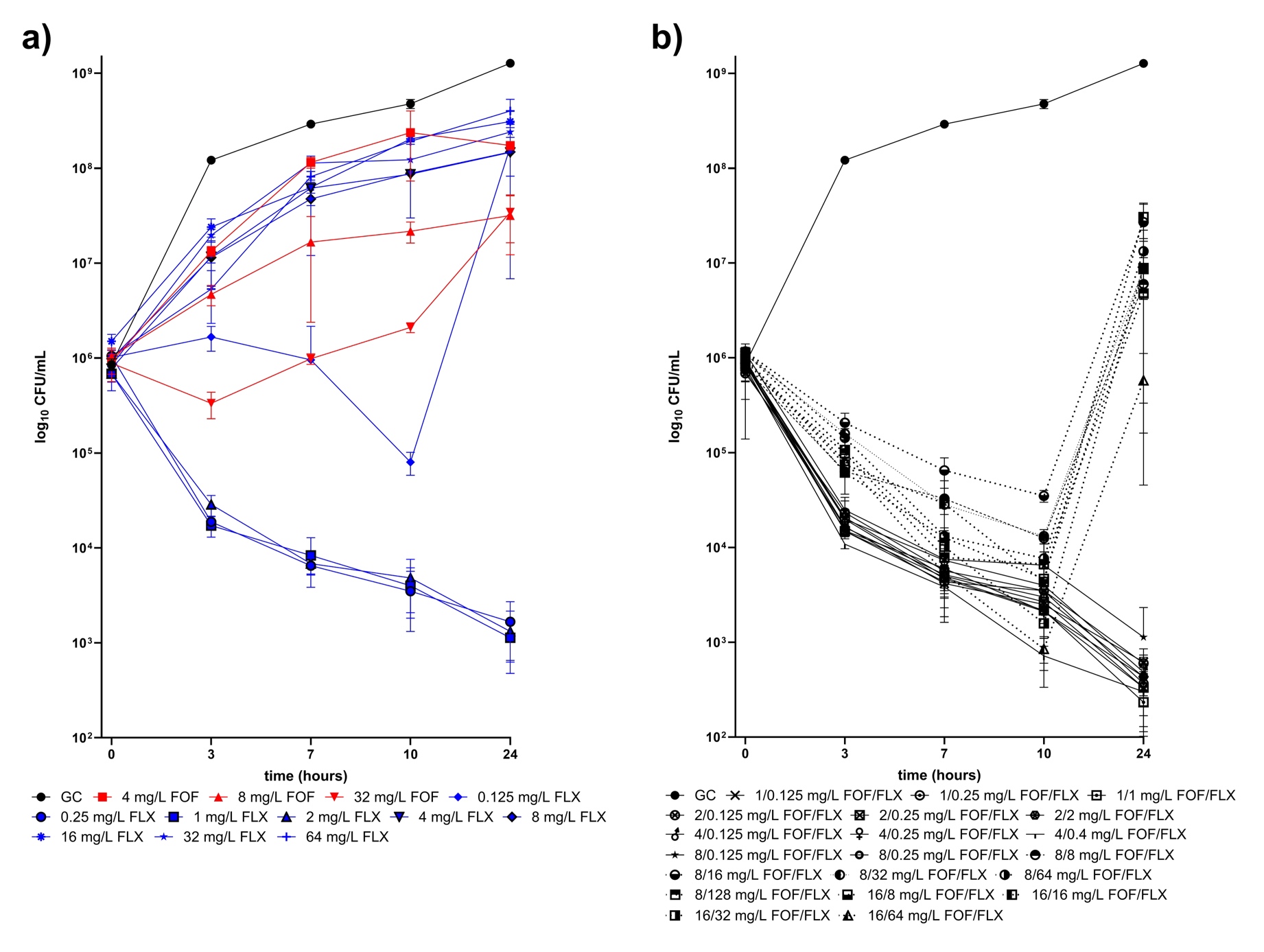


***DNA Extraction and Sequencing***

The extracted and diluted DNA showed high purity with 260/280 ratios of 1.98 ± 0.04 SD and 260/230 ratios of 2.36 ± 0.06 SD. The Q30 of the raw sequencing output was 74% indicating an error rate less than 1:1000. The cluster density of the run was 1443 K/mm² and 88.43% of the reads passed filters. The coverage of the de novo assemblies was 91x.


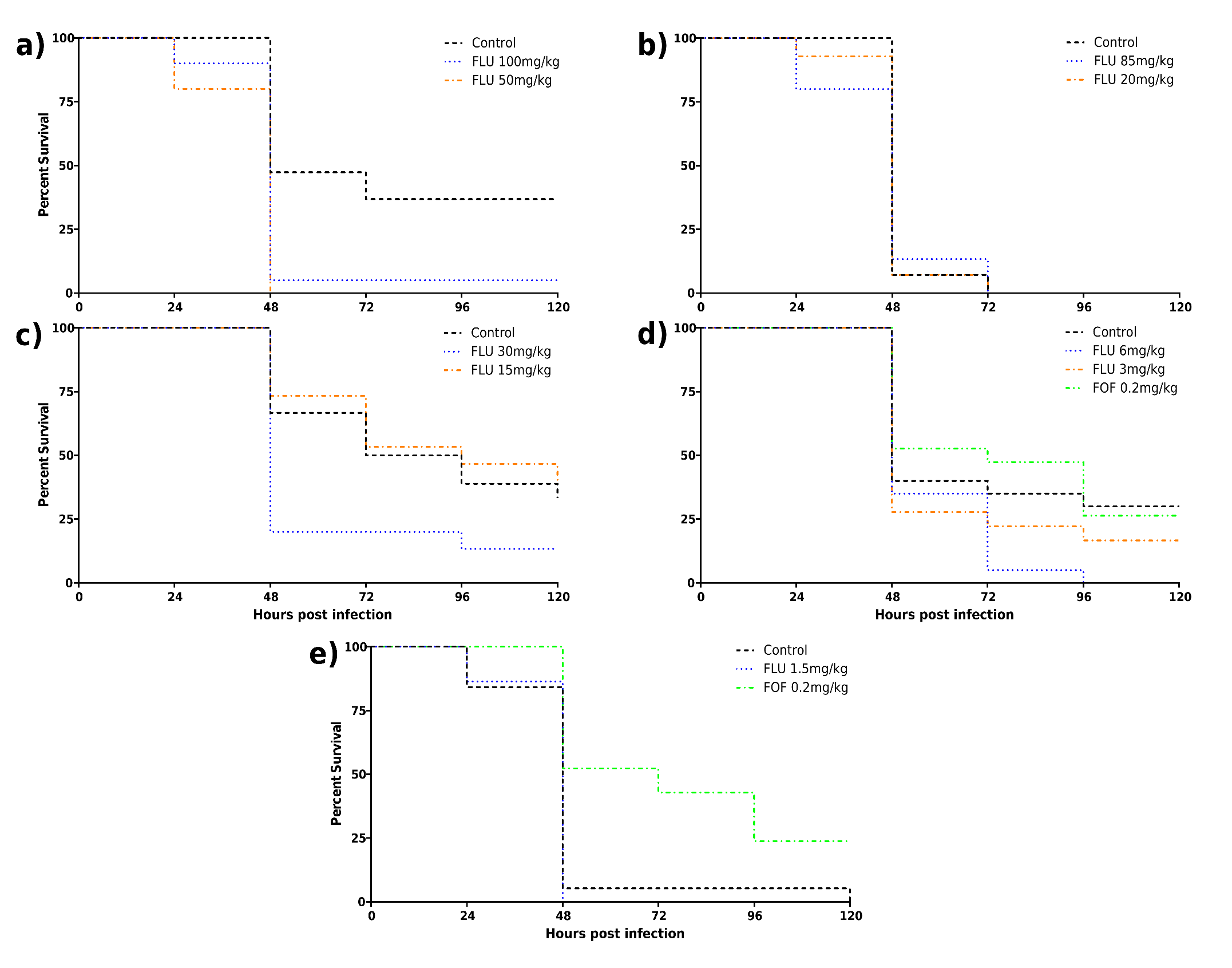


**Figure S5 a-e)** Survival curve dose-finding experiments with *G. mellonella* larvae infected with methicillin-suceptible *S. aureus* ATCC 29213. Flucloxacillin was used at various dosages: **a)** 100mg/kg (n= 20) and 50mg/kg (n= 20), **b)** 85mg/kg (n= 15) and 20mg/kg (n= 14), **c)** 30mg/kg (n= 15) and 15 mg/kg (n= 15), **d)** 6mg/kg (n= 20) and 3mg/kg (n= 18) and **e)** 1.5mg/kg (n= 22). In experiment **d)** and **e)** low-dose fosfomycin (FOF) was used as a comparator drug using groups of 19 and 22 larvae, respectively. Larvae in the control groups were infected but received only sterile PBS (a) n= 19, b) n= 14, c) n= 18, d) n= 20, e) n= 19).

**
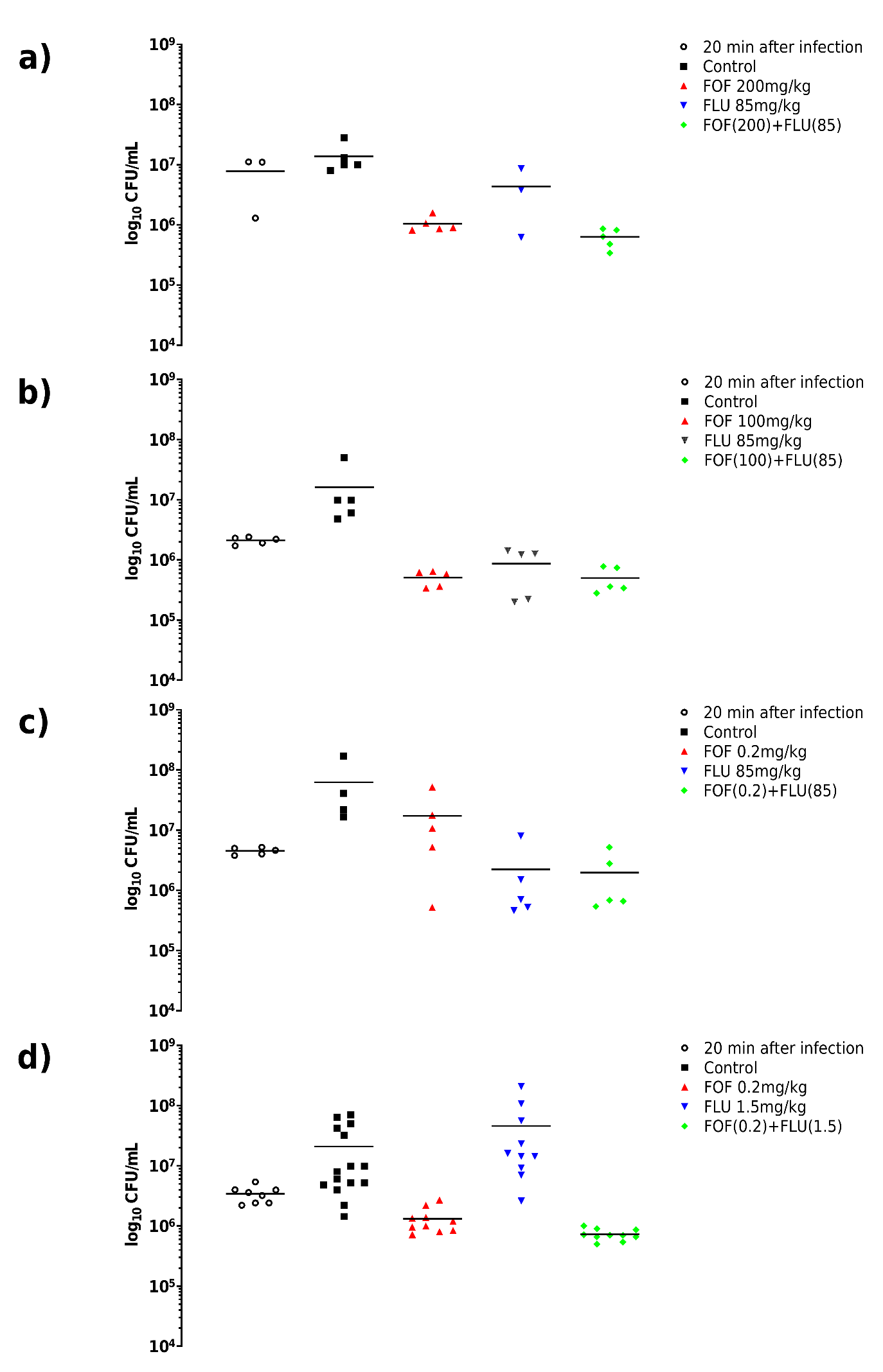
Figure S6 a-d)** Mean and individual bacterial counts in log_10_ colony-forming units/mL (CFU/mL) of *G. mellonella* larvae determined 24 hours after infection with methicillin-susceptible *S. aureus* ATCC 29213. Treatment groups received various doses of flucloxacillin (FLU), fosfomycin (FOF), or combinations of both. The control groups received sterile PBS only. Baseline bacterial counts were determined 20 minutes after infection.

References:

[1] Galore T. B.B.-T. Trim Galore v0.6.5 . Https://WwwBioinformaticsBabrahamAcUk/Projects/Trim_galore/ n.d.

[2] Prjibelski A, Antipov D, Meleshko D, Lapidus A, Korobeynikov A. Using SPAdes De Novo Assembler. Curr Protoc Bioinformatics 2020;70. https://doi.org/10.1002/cpbi.102.

[3] Gurevich A, Saveliev V, Vyahhi N, Tesler G. QUAST: Quality assessment tool for genome assemblies. Bioinformatics 2013;29:1072–5. https://doi.org/10.1093/bioinformatics/btt086.

[4] Koboldt DC, Zhang Q, Larson DE, Shen D, McLellan MD, Lin L, et al. VarScan 2: Somatic mutation and copy number alteration discovery in cancer by exome sequencing. Genome Res 2012;22:568–76. https://doi.org/10.1101/gr.129684.111.

[5] Langmead B, Salzberg SL. Fast gapped-read alignment with Bowtie 2. Nat Methods 2012;9:357–9. https://doi.org/10.1038/nmeth.1923.
